# Redox signaling in pathophysiology of hypertension

**DOI:** 10.1186/1423-0127-20-69

**Published:** 2013-09-18

**Authors:** Miroslava Majzunova, Ima Dovinova, Miroslav Barancik, Julie YH Chan

**Affiliations:** 1 Institute of Normal and Pathological Physiology, Slovak Academy of Sciences, Sienkiewiczova 1, 813 71 Bratislava, Slovakia; 2 Institute for Heart Research, Slovak Academy of Sciences, Bratislava, Slovakia; 3 Center for Translational Research in Biomedical Sciences, Kaohsiung Chang Gang Memorial Hospital, Kaohsiung, Taiwan

**Keywords:** Radical signaling, Antioxidant response, Kinase pathway, Transcriptional factor, Reactive oxygen species, Hypertension, Redox-sensitive signaling

## Abstract

Reactive oxygen species (ROS) are products of normal cellular metabolism and derive from various sources in different cellular compartments. Oxidative stress resultant from imbalance between ROS generation and antioxidant defense mechanisms is important in pathogenesis of cardiovascular diseases, such as hypertension, heart failure, atherosclerosis, diabetes, and cardiac hypertrophy. In this review we focus on hypertension and address sources of cellular ROS generation, mechanisms involved in regulation of radical homeostasis, superoxide dismutase isoforms in pathophysiology of hypertension; as well as radical intracellular signaling and phosphorylation processes in proteins of the affected cardiovascular tissues. Finally, we discuss the transcriptional factors involved in redox-sensitive gene transcription and antioxidant response, as well as their roles in hypertension.

## Review

### Introduction

Living organisms can reduce oxygen to water *via* aerobic oxidative phosphorylation. This significantly shapes cellular metabolism and energy production. The associated oxygen intake results in the formation of toxic reactive oxygen species (ROS) in the mitochondrial respiratory chain. These radicals are electron donors and can damage DNA, RNA, proteins, and lipids. They can also propagate deleterious reactions throughout cells and tissues resulting in cell death and apoptosis [1]. In addition, ROS can alter gene expression by modulating the activation of transcription factors, with subsequent influence on downstream target proteins that regulate cellular functions such as cell growth and differentiation, modulate production and degradation of extracellular matrix, inactivate nitric oxide (NO) functions, and stimulate multiple kinases and proinflammatory gene expressions. The ROS play an important role in the development of cardiovascular diseases such as hypertension, heart failure, atherosclerosis, diabetes, and cardiac hypertrophy. Increased production of oxidants, reduced NO bioavailability and reduced capacity of antioxidants in the vascular system and kidneys are involved in these diseases [2].

Studies of the role of ROS and reactive nitrogen species (RNS) in signal transduction have grown in number during the past decade. The main interest in the field is to recognize the specific targets and chemical reactions involved in the signaling pathways triggered by ROS and RNS as second messenger molecules. It was observed that cysteine residues in the thiolate (ionized) form are present in several classes of signaling proteins, and these can be the specific targets for reactions with hydrogen peroxide (H_2_O_2_) and RNS. These findings suggest that in many signaling events where ROS and RNS take part, enzymatic catalysis is more likely to be involved than a non-enzymatic reaction [3]. As such, aberrant redox-sensitive signal transduction is involved in pathophysiology of hypertension [4-7]. In this review, it is not our intention to provide a detailed survey on literature of ROS in pathogenesis of hypertension, since many comprehensive reviews in this aspect are available [8-12]. We shall focus on different sources of ROS in the cardiovascular systems and summarize current knowledge on the redox-sensitive signaling in pathophysiology of hypertension.

### ROS generation

**Figure 1:**
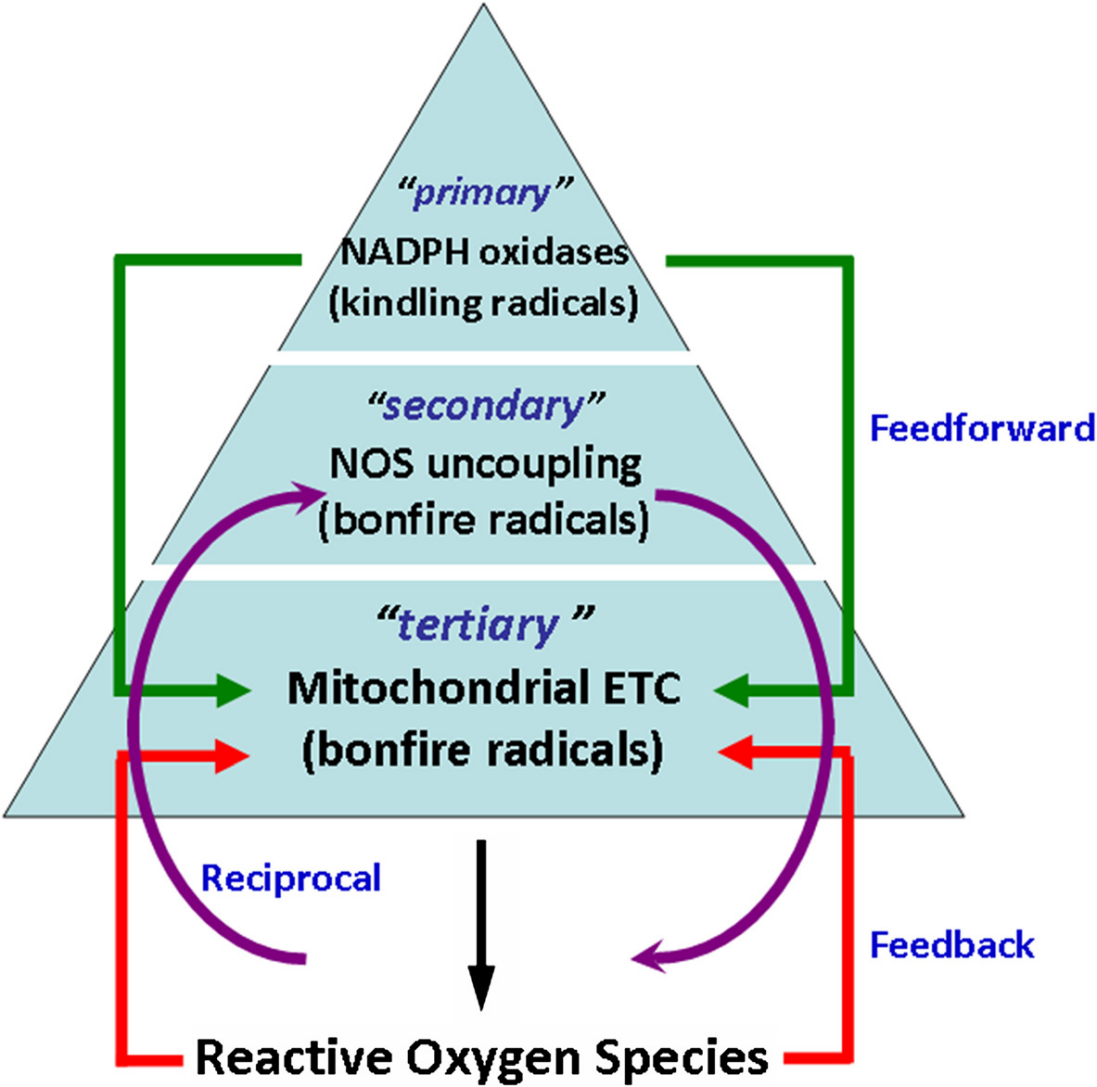
ROS sources and their function according to “kindlig-bonfire” theory.

Several mechanisms or pathways are responsible for the production of free radicals in the cell [13,14]. A paradigm has arisen over the past several years whereby small amounts of radicals (so called “kindling radicals”) can lead to formation of peroxynitrite, which oxidizes tetrahydrobiopterin (BH_4_), leading to uncoupling of the endothelial NO synthase (eNOS) and to formation of large amounts of “bonfire” radicals [15]. As a result, production of one free radical can lead to formation of other radicals through a sequential chain reaction. According to this “kindling-bonfire” theory of vascular oxidative stress, there are primary, secondary and tertiary sources of ROS. In general, nicotinamide adenine dinucleotide phosphate (NADPH) oxidase acts as the primary source of superoxide anions (O_2_^-•^) and H_2_O_2_ in the vessel wall. NADPH oxidase-derived ROS then act as “kindling” and activate secondary (uncoupled eNOS, xanthine oxidase), and tertiary (mitochondrial) sources of ROS, which contribute to the “bonfire” of radicals and oxidative stress seen at later stages of diseases [16] (Figure 1).

### NADPH oxidase-derived ROS and hypertension

ROS are derived from many sources in different cellular compartments. In the vascular smooth muscle cells and endothelial cells, NADPH oxidase acts as the primary source and is particularly important in pathophysiology of hypertension (Figure 1). In the vascular system, ROS production *via* the NADPH oxidase is triggered by stimulation of neurohumoral vasoconstrictor agents, such as angiotensin II (Ang II), endothelin-1 (ET-1) and norepinephrine (NE). The action of Ang II through angiotensin type 1 (AT_1_) receptors plays an important role in vasoconstriction. Activation of AT1 receptors results in induction of a number of ROS-producing events in the cell. Infusion of Ang II to normotensive rats stimulates the production of O_2_^-•^ by NADPH oxidase in vessels and induces pressor responses [17]. NADPH oxidase can also be activated by aldosterone and ET-1 [18]. ET-1, the main endothelin form in the endothelium, is a potent vasoconstrictor produced in various vascular tissues including the endothelium. When delivered in high concentrations, ET-1 acts as a vasoconstrictor and is able to alter arterial pressure. The effects of ET-1 are mediated by two isoforms of receptors, ET_A_ and ET_B_. Activation of ET_A_ receptors causes contraction of blood vessels *via* activation of the NADPH oxidase and generation of ROS; activation of ET_B_ receptors leads to relaxation of vessels [19]. In cardiomyocytes, the production of ROS is stimulated also by NE *via* α_1_-adrenergic receptors (α_1_-AR). α_1_-AR, AT_1_, and ET-1 receptors are known to activate NADPH oxidase through the G-protein, resulting in the production of ROS [20]. Significance of the NADPH oxidase-derived ROS in pathogenesis of hypertension was comprehensively discussed in several recent reviews [12,21-23].

### NOS uncoupling and hypertension

Under physiological conditions, NOS, in the presence of substrate L-arginine and co-factor BH_4_, produces NO. When BH_4_ levels are reduced, stability of NOS dimer formation is altered such that the oxidase domain yields molecular uncoupling and the catalytic activity becomes functionally 'uncoupled’ [12]. Enzymatic reduction of molecular oxygen by eNOS no longer couples to L-arginine, resulting in the generation of deleterious O_2_^-•^ rather than protective NO [24]. This eNOS uncoupling (Figure 1) contributes to the increased ROS production and endothelial dysfunction observed in various vascular diseases [25,26], including hypertension [27]. Detailed discussion on significance of eNOS uncoupling in pathogenesis of hypertension is beyond the scope of this review and readers are recommended to read other reviews [26-28] for detailed discussion.

### Mitochondrial ROS and hypertension

Mitochondrial electron transport chain is another important source of ROS generation. Any damage at enzyme complexes of the mitochondrial respiratory chain leads to dysfunction of the mitochondrial respiration with subsequent transfer of electrons to molecular oxygen increasing the mitochondrial O_2_^-•^ and H_2_O_2_ formation [29]. In addition, increased mitochondrial peroxynitrite formation leads to nitration and inactivation of mitochondrial antioxidant, manganese superoxide dismutase (MnSOD) [30], which results in the impaired breakdown of mitochondrial O_2_^-•^ and further exacerbating in ROS production. Finally, cytosolic ROS, *via* opening of the redox-dependent mitochondrial ATP-sensitive potassium channels [31] and changes in the mitochondrial membrane potential [32], stimulates the mitochondrial ROS formation (Figure 1). Reduced capacity of the mitochondrial respiratory chain was observed in the brain stem of the spontaneously hypertensive rats (SHR) and in neurogenic hypertension induced by Ang II [33].

Mitochondria are integral component in energy metabolism, ROS generation, and cell signaling, thus play an important role in oxidative stress leading to the development of many cardiovascular conditions, including hypertension. The functional significance of mitochondria-derived ROS, particularly in vascular cells, has recently received a great attention. For example, hyperinsulinemia induces migration and proliferation of vascular smooth muscle cells *via* the increase in mitochondrial ROS [34]. Interestingly, production of free radicals and impairment of mitochondrial function in young SHR has been affected after long-term treatment with AT_1_R blocker, losartan. Glutamate-supported respiration, the rate of ATP production, and concentrations of coenzyme Q9 (CoQ9), coenzyme Q10 (CoQ10), and alpha-tocopherol have all been reported to improve mitochondrial dysfunctions and promote antihypertension in SHR after the therapy [35].

During acute hypoxia, O_2_^-•^ released from mitochondrial Complex III of smooth muscle cells diffuses into the cytosol and triggers increases in intracellular calcium and causes acute hypoxic pulmonary vasoconstriction [36,37]. Moreover, mitochondrial ROS cross-talk with NADPH oxidase markedly augments redox responses to Ang II in the vascular smooth muscle cells [38]. This cross-talk between mitochondrial ROS and the NADPH oxidase in the rostral ventrolateral medulla (RVLM), key nucleus for the generation of sympathetic outflow from the brain stem, was recently demonstrated to play an active role in manifestation of high arterial pressure in SHR [39].

### Redox-sensitive intracellular signaling in hypertension

The term “redox signaling” describes a process in which physiological levels of ROS induce modifications to proteins that are discrete, site-specific, and reversible [40]. Evidence emerged over the past 2 decades suggests that ROS modulate the activity of a vast array of intracellular proteins and signaling pathways, and this redox signaling is spatially and temporally regulated to generate specific cellular effects under health and disease.

**Figure 2:**
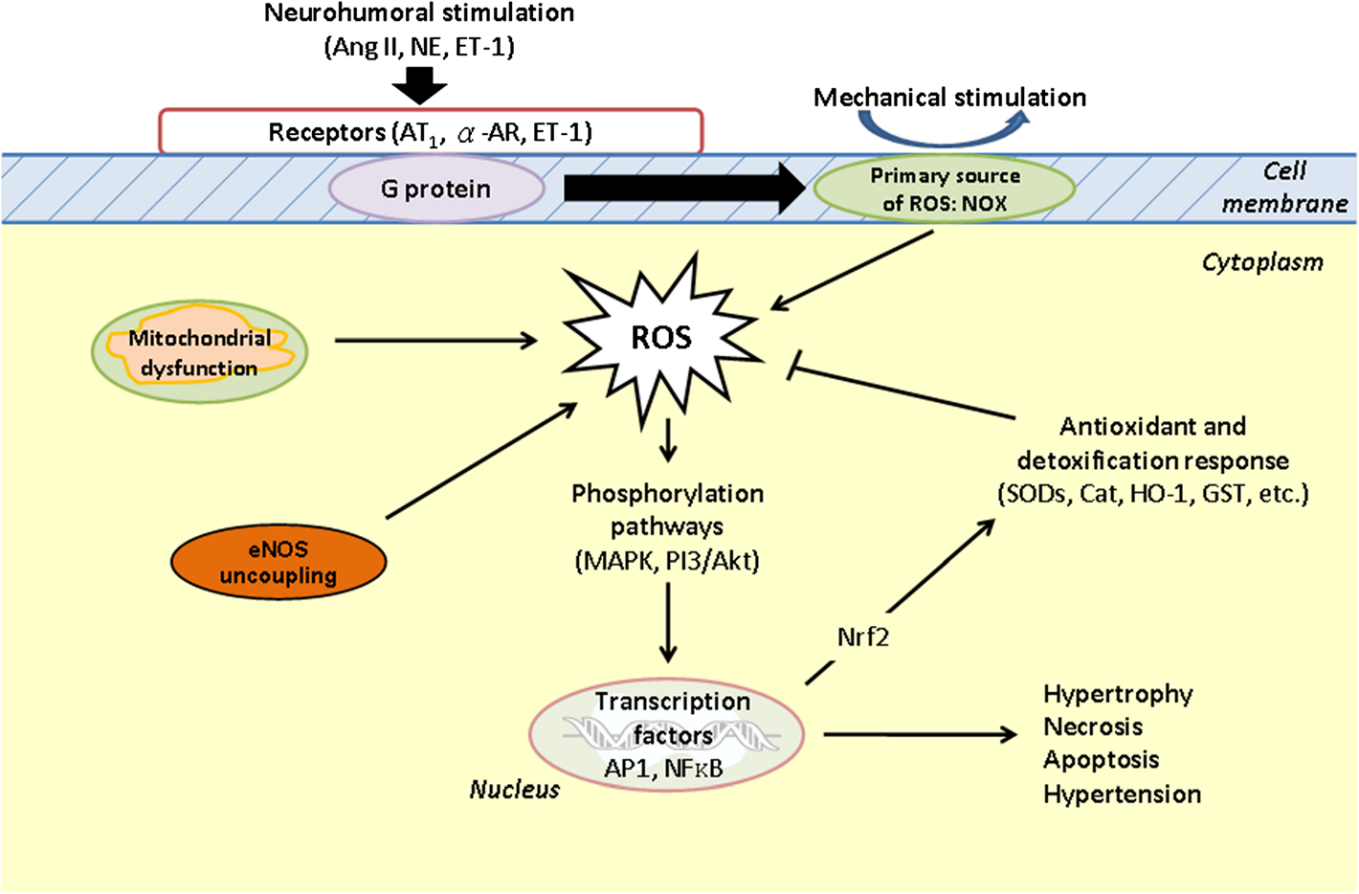
Redox-sensitive intracellular signaling in hypertension.

Until recently, ROS were considered only as harmful molecules. However, recent studies suggest that ROS may serve as an important physiological regulator of intracellular phosphorylation signaling pathways. The specific effects of ROS are largely modulated by covalent modification of specific cysteine residues, which were discovered in redox-sensitive proteins. Oxidation of these reactive cysteine residues can lead to reversible modification of enzyme activity. There is evidence that the ROS regulate various physiological parameters in response to stimulation with growth factors to generate inflammatory response. Disruption of normal ROS signaling may lead to several diseases in humans, including hypertension [41]. In hypertension, neurohumoral stimuli such as Ang II, NE, and ET-1 activate receptors located on cell membrane, namely AT1, α-AR, and ET receptors. The function of these receptors is coupled to G proteins, which activate the source of ROS, NADPH oxidase. The activated NADPH oxidase will produce ROS (e.g., O_2_^-•^), and these, in turn, activate cell phosphorylation pathways: the mitogen-activated protein kinases (MAPKs), tyrosine kinases, and phosphoinositol-3-kinase/Akt kinase (PI3K/Akt). The activated phosphorylation pathways activate transcription factors such as activated protein-1 (AP-1), p53, nuclear factor kappa B (NFκB), and nuclear E2-related factor 2 (Nrf2), which stimulate transcription of genes after moving into the nucleus. Proteins encoded by these target genes in turn mediate cellular consequences leading to changes in the phenotypes, such as hypertrophy, inflammation, necrosis and apoptosis of cells and, on the other hand, stimulate the production of antioxidants involved in antioxidant defense [17,20,41] (Figure 2).

Despite the fact that cells of different systems involved in the regulation of blood pressure perform different functions, the redox signaling is by and large very similar and unveil no apparent differences among these types of cells. NADPH oxidases are primary resources of ROS in the endothelial cells, vascular smooth muscle cells, cardiomyocytes, cells of kidneys, as well as cardiovascular neurons (regions such as the circumventricular organs, paraventricular nucleus of the hypothalamus, nucleus tractus solitarii and RVLM) in the brain [10,12,21,42-44]. Ang II is important activator of NADPH oxidases and stimulator of ROS production in all types of cells [20,42-46]. Production of ROS is stimulated by shear and mechanical stress in the vascular smooth muscle cells and cardiomyocytes, respectively. In addition, MAPKs are responsible for major effects of ROS such as proliferation, hypertrophy, and apoptosis in these cells [20,42,45-47]. The same signaling is also proposed to mediate the increase in sympathetic nerve activity, vasopressin release and drinking behavior induced by Ang II (Table 1).

**Table 1:** Redox-sensitive intracellular signaling in different cells

**Type of cell**	**Stimuli inducing production of ROS**	**Phosphorylation pathways**	**Transcription factors**	**Effects of ROS in target cells**	**Ref.:**
**Endothelial cells**	Ang II	ERKs, JNKs	NFκB	Apoptosis	[45,46]
		p38-MAPKs		Dysfunction	
**Vascular smooth muscle cells**	PDGF, phenylephrine, thrombin	MAPKs	NFκB	Proliferation	[45-47]
	Ang II	p38-MAPK	NFκB?	Hypertrophy, migration	
	Ang II	PI3K/Akt			
	ET-1				
	Shear stress				
**Cardiomyocytes**	Ang II	MAPKs	NFκB	Hypertrophy, inflammation, necrosis, apoptosis	[20]
	ET-1	PI3K/Akt	AP-1		
	NE		p53		
	Mechanical stress				
**Central cardiovascular neurons**	Ang II	p38-MAPK,	??	Increased sympathetic nerve activity, vasopressin release, drinking behavior	[43,44,48]
		ERK 1/2			
		PLC/IP3 ?			
		(or NO ?)			
**Cells of kidney**	Ang II	MAPKs		Augmentation of epithelial-mesenchymal transition, mesangial cells apoptosis, hypertrophy	[42]
	Aldosterone	ERK1/2			
	Chemokines				

#### ***Mitogen-activated protein kinases***

The MAPK family includes serine-threonine protein kinases that play an important role in the transmission of extracellular signals from cell membrane to the nucleus. Activation of MAPKs and signal transduction depends on series of phosphorylating events that allow interaction of multiple signaling pathways. MAPK phosphorylation cascades can be negatively regulated by MAP phosphatases that dephosphorylate/deactivate individual MAPKs [49]. The MAPK pathways are involved in the regulation of cell proliferation, differentiation, transformation and death, as well as in vasoconstriction [50]. Three main subgroups of MAPKs were discovered in the vascular system of mammals: (a) extracellular signal-regulated kinases (ERKs); (b) c-Jun N-terminal kinases (JNKs); (c) p38-MAPK. MAPK pathways; all can be activated by many extracellular and intracellular stimuli such as growth factors (Ang II, vascular endothelial growth factor, platelet-derived growth factor) [51], inflammatory cytokines, and cellular stress [52]. Oxidative stress is a particular type of such stress that induces activation of MAPK pathways [53]. Studies have shown that increased production of ROS is responsible for activation of redox-sensitive p38-MAPK, which might be involved in the functional and structural changes associated with hypertension [54]. In SHR, p38-MAPK is essential for collagen synthesis and mediates the growth of vascular smooth muscle cells [51]. The major source of ROS involved in kinase activation is NADPH oxidase. Although the precise mechanisms of redox-sensitive MAPK activation are not fully understood, the likely mechanisms include: (a) oxidation of cysteine residues of receptors for growth factors and cytokines; (b) oxidative modification of intracellular kinases involved in the MAPK signaling cascade; (c) deactivation and degradation of MAPK phosphatases that maintain the MAPK pathway in the inactive state [52].

#### ***Tyrosine kinases***

Activation of tyrosine kinases by ROS plays an important role in the remodeling of the cardiovascular system associated with hypertension. In vascular smooth muscle cells and other cells, the ROS activate non-receptor tyrosine kinases such as Janus kinase 2 (JAK2), proline-rich tyrosine kinase 2/cell adhesion kinase (PYK2/CAK) and Src tyrosine kinase, or the receptor tyrosine kinases, such as the receptor for epidermal growth factor (EGF) [55-57]. Particular attention is paid to the EGF receptor and JAK2 tyrosine kinase in smooth muscle cells. The EGF receptor is activated by Ang II *via* ROS. Its activation is necessary for activation of the ERKs and for the growth of vascular smooth muscle cells [58]. The JAK2 tyrosine kinase is also activated by Ang II and ROS and is essential for the induction of cytokines and thrombin-induced heat-shock protein in vascular smooth muscle cells and for subsequent induction of inflammatory response [59].

#### ***Akt kinases***

The Akt kinase (or protein kinase B) is a serine/threonine kinase that plays an important role in many cellular processes and its activation in the PI3K/Akt pathway depends on the upstream PI3K. The activated Akt kinase phosphorylates various downstream effector molecules, such as glycogen synthase kinase-3, procaspase-9, transcription factors AP-1 and E2F [60], and is thus involved in modulation of cellular functions. The Akt kinase cascade is implicated in the processes of cell survival and its anti-apoptotic effects are mediated by phosphorylation of several proteins involved in apoptosis, such as the pro-apoptotic Bad protein. In addition to its effect on cell survival, Akt kinase plays an important role in protein synthesis, in the development of hypertrophy [61], and its activation was found to play a role in infarct size limiting mechanisms following cardiac ischemic injury [62]. Dysregulation of Akt leads to diseases of major unmet medical need such as cancer, diabetes, cardiovascular and neurological diseases [63].

The PI3K/Akt kinase signaling pathway is also involved in the metabolic control and regulation of glucose transport and glycogen synthesis. The Akt kinase can be activated by a variety of growth stimuli: the platelet-derived growth factor, EGF, insulin, thrombin, and the nerve growth factor (NGF) [64]. It is also known that the oxidative stress induced by ROS is accompanied by activation of Akt kinase. Akt kinase activation by ROS and Ang II has been documented in smooth muscle cells [61]. In myocardium, Akt kinase has been considered as a reperfusion injury salvage kinase, counteracting oxidative stress-induced cell damage through a compensatory protective pathway [65]. In the RVLM of brain stem, ROS activates PI3K/Akt signaling *via* oxidation and phosphorylation of the phosphatase and tensin homolog deleted on chromosome 10, which is a negative regulator of PI3K signaling [66]. This redox-sensitive overactivation of PI3K/Akt signaling contributes to neural mechanisms of hypertension in SHR. Changes in Akt kinase activation were also observed in the doxorubicin-treated normotensive Wistar rats. Eight weeks after the treatment, the systolic blood pressure is increased, alongside with an increase in matrix metalloproteinase-2. These changes are accompanied by activation of Akt kinase, an increase in ROS accumulation, inhibition of SOD activity, induction of inducible NOS protein expression and caspase-3 activation [67]. In human endothelial cells, Akt kinase was found to phosphorylate the eNOS at serine-1177, and this is connected with eNOS activation and production of NO. The activation of eNOS through the Akt kinase was observed also by shear stress and insulin [60].

#### ***RhoA/ROCK pathway***

The Rho-associated protein kinase (ROCK) is a serine/threonine kinase. For its activity, ROCK requires the binding of the small G-protein RhoA. The RhoA/ROCK signaling pathway is stimulated by many agonists such as Ang II, thrombin, ET-1, and NE. Increased activity of this pathway was observed in atherosclerosis and pulmonary hypertension, and this signaling has been implicated in many major cardiovascular diseases such as hypertension, heart failure, and myocardial infarction. A role of this pathway in the regulation of several cellular functions such as gene expression, contraction, migration, and proliferation has been reported [68]. Moreover, it has been demonstrated that this pathway downregulates eNOS phosphorylation through inhibition of Akt kinase [69], and is involved in the modulation of eNOS gene expression by affecting the stability of its mRNA [70]. In vascular smooth muscle cells, the effects of RhoA/ROCK pathway on the decreased NO production were associated with increased vascular contractility. Moreover, inhibitors of RhoA or ROCK can decrease the systolic blood pressure in hypertensive rats [71]. Essential hypertension in human is associated with increased Rho/Rho-kinase-dependent mechanisms and ROCK activity [72].

### Redox-sensitive transcription factors and nuclear factors in hypertension

There are several transcription factors, including AP-1, p53, NF-κB, Nrf2, whose activity is sensitive to oxidative stress. AP-1 was first identified as a transcription factor that binds to the *cis*-element of the methallothionein human genes encoding proteins that are involved in cell proliferation and apoptosis in response to various stimuli, including the oxidative stress. Redox-sensitive upregulation of AP-1, c-*jun* or c-*fos* have been observed in the RVLM of the hypertensive animals [73]. In deoxycorticosterone acetate-induced hypertension, elevated inflammation and tissue damage in cardiac and vascular muscle cells are attributed to oxidative stress-associated activation of AP-1 [74]. In this animal model of hypertension, dysfunction of circulating endothelial progenitor cells is related to ET_A_ receptor-mediated and oxidative stress-associated overexpression of p53 [75]. In addition, NF-κB activation by ROS induces the cytosolic and mitochondrial oxidative stress and tissue injury that contribute to renal dysfunction observed in SHR [76].

AP-1 binds to the antioxidant response element (ARE) of target genes, where Fos and Jun proteins may heterodimerize Nrf2 in the presence of electrophiles and oxidants [1]. Activation of gene transcription *via* the ARE is mediated primarily through the Nrf2 transcription factor. Under normal conditions, the activity of Nrf2 is negatively regulated by protein Kelch-like ECH associated protein 1 (Keap1) that binds to Nrf2 and keeps it in the cytoplasm. Exposure to xenobiotics, NO and/or ROS induce the transcription of Keap1/Nrf2/ARE‒dependent genes whose functional products protect against a wide array of subsequent challenges [77]. Free Nrf2 is translocated into the nucleus, where it binds ARE enhancer and activates ARE-dependent transcription of antioxidant stress genes to trigger the cellular defense response [78,79]. Therefore, redox-sensitive activation of nuclear enhancer Nrf2 serves as a self-defense feedback mechanism to prevent sustained oxidative damage.

In addition to enzymatic degradation, detoxification of ROS and electrophiles is also important in protecting cells from oxidative damage. Genes regulated through the ARE are involved in the production of antioxidants that directly inactivate ROS (such as catalase, superoxide dismutase), detoxify xenobiotics (glutathione-S-transferase, thioredoxin, NAD(P)H quinone oxidoreductase) and affect the production of glutathione and phase-II detoxification enzymes (e.g., γ-glutamate cystein ligase, heme oxygenase-1). All these genes are highly sensitive to increases in oxidative stress [80]. Moreover, several redox-sensitive kinases, such as protein kinase C, PI3K/Akt, MAPKs, casein kinase-2 [81], are involved in the regulation of Nrf2 activity. These kinases regulate the stability and localization of Nrf2 by its phosphorylation on serine or threonine residues in a cell type-specific manner [80].

Emerging evidence indicates that activation of Nrf2/ARE signaling differs during acute versus chronic stress conditions. During acute oxidative stress, oxidized molecules modify multiple cysteine residues in Keap1, causing Nrf2 to be released from connection with Keap1. Nrf2 is translocated into the nucleus and induces transcription *via* the ARE genes [78,82]. During a prolonged oxidative stress, the activity of Nrf2 is turned off by glycogen synthase kinase 3β (GSK3β). The activated GSK3β phosphorylates the Fyn tyrosine kinase, causing its relocation to the nucleus [83]. Fyn phosphorylates Nrf2, which leads to expulsion of Nrf2 from the nucleus, Nrf2 binds to Keap1 and is degraded in consequence [84]. This mechanism leads to attenuation of antioxidant and detoxificant response in long term oxidative stress. Only recently the involvement of this redox-sensitive regulation of Nrf2-ARE cascade gained interest on its role in pathogenesis of oxidative stress-associated hypertension. The mammalian Nox4 of the NADPH oxidase possesses antioxidant activity through Nrf2-dependent upregulation of antioxidant heme oxygenase-1 (HO-1) [85]. Catalase overexpression ameliorates maternal diabetes-induced perinatal programming and development of hypertension in adulthood, mediated, at least in part, by triggering the Nrf2-HO-1 defense system [86]. On the other hand, Nrf2-deficient mice show reduced pulmonary expression of antioxidant glutathione, and this renders them highly susceptible to hyperoxia-induced oxidative lung injury [87].

### SOD isoforms and management of hypertension

For the cell to maintain ROS homeostasis there is a delicate balance existing between the production and degradation of the radicals [88,89]. The later involves activation of SOD isoforms, which are the first natural antioxidant defense proteins that improve redox balance. There are three SOD isoforms, namely the cytosolic copper-zinc SOD (CuZnSOD, or SOD1), mitochondrial manganese SOD (MnSOD, or SOD2), and the extracellular SOD (ecSOD, SOD3). They are the products of different genes, but catalyze the same reaction for dismutation of O_2_^-•^ to H_2_O_2_[90]. The SOD function as a signaling molecule in blood vessels and cardiomyocytes have been discussed [15,91]. Applications of antioxidant gene therapies based on the SOD genes, HO-1 gene, or glutathione peroxidase gene in cardiovascular diseases have been presented and summarized [92]. Application of individual SOD isoform in the therapy of hypertension has been described [93].

Zimmerman et al. [94] were the first to report *in vivo* gene delivery of adenoviral vectors encoding SOD1. They found that both superoxide production from Ang II infusion and the induced hypertension were prevented by overexpression of CuZnSOD. A causal relationship between biochemical correlates of oxidative stress and neurological hypertension was established after a gene transfer by microinjection of adenovirus encoding SOD1, SOD2 or catalase into the RVLM, which promotes a long-term reduction of blood pressure in SHR [95]. In consistent to the findings, a gene therapy based on SOD1 gene transduction by a bacterial gene delivery system results in blood pressure decrease, regulation of SOD and NOS activities, and a protection against oxidative stress [96].

SOD2 is present mainly in the mitochondria of endothelial cells and less in mitochondria of vascular smooth muscle cells [90]. Mitochondrial oxidative damage is one of the main pathogenic cellular mechanisms involved in pathogenesis of hypertension, making the selective targeting of the mitochondria with potent antioxidants a promising approach in the therapeutic strategy of these diseases. Overexpression of SOD2 reduces oxidative stress in mitochondria and promotes antihypertensive effects [90]. Preservation of the mitochondrial electron transport capacity in RVLM with a highly mobile electron carrier, CoQ10, reduces arterial pressure in SHR and attenuates the pressor response of normotensive Wistar-Kyoto rats to Ang II infusion [7,33]. Transgenic mice with SOD2 overexpression attenuates hypertension induced by Ang II [97]. This situation is similar to mice treated with mitochondrial-targeted antioxidant mitoTEMPO, while non-target antioxidant TEMPOL was not effective [98]. Reduction in oxidative stress by intravenous transduction of cDNA of the SOD1 and SOD2 genes leads to a decrease in blood pressure, reversal of neuronal NOS gene upregulation in the left ventricle, and a decreased mitochondrial damage in adult SHR [99].

SOD3 is located in the extracellular space and binds to the cell membrane through a heparin-binding domain. It is the second most abundant SOD isoform in blood vessels and is produced mainly by smooth muscle cells. SOD3 is very important in endothelium-mediated vasodilation because of its location in the interstitium between endothelial cells and vascular smooth muscle cells. It enables safe diffusion of NO from endothelial cells into smooth muscle cells [100]. EcSOD-deficient mice have significantly higher degree of hypertrophy, ventricular dilation and myocardial fibrosis [101]. Studies using overexpression revealed the protective effects of SOD3 in the blood vessels. Gene transfer of SOD3-encoding cDNA reduces superoxide levels in blood vessels and decreases arterial pressure in SHR [102].

## Conclusions

Radical signaling offers several possibilities and strategies of modulating ROS homeostasis in the management of hypertension. Individual SOD isoforms can be influenced using gene transduction and SOD mimetics. Other strategies include inhibition of NADPH oxidase activity, preservation of mitochondrial electron transport chain capacity, increases in BH_4_, modulation of the signaling and phosphorylation pathways, such as MAPK, Akt kinase, and RhoA/ROCK signaling pathways. All these possibilities and strategies have been reported to alleviate ROS accumulation and to protect against oxidative stress to a different degree in the animal models of hypertension. In addition, modulation of the redox-sensitive transcription factors, as well as stimulation of antioxidant response through the Nrf2-ARE pathway may also prove to be a useful strategy in future. All these possibilities and strategies should be further studied in the search for new therapeutical approaches in management of hypertension.

## Abbreviations

Akt kinase: Serine/threonine protein kinase; Ang II: Angiotensin II; AP-1: Activator protein 1; ARE: Antioxidant response element; AT1R: Angiotensin type 1 receptor; α1-AR: alpha_1_-adrenergic receptors; BH4: Tetrahydrobiopterin; CoQ: Coenzyme Q; Cu/Zn SOD: Cupper/zinc superoxide dismutase; ecSOD: extracellular superoxide dismutase; EGF: Epidermal growth factor; eNOS: endothelial nitric oxidase synthase; ERK: Extracellular signal-regulated kinase; ET-1: Endothelin-1; GSK-3β: Glycogen synthase kinase-3β; H2O2: Hydrogen peroxide; HO-1: Heme oxygenase-1; JAK2: Janus kinase 2; JNK: c-Jun N-terminal kinase; Keap1: Kelch-like ECH associated protein 1; NADPH oxidase: Nicotinamide adenine dinucleotide phosphate oxidase; MAPK: Mitogen activated protein kinase; MnSOD: Manganese superoxide dismutase; NE: Norepinephrine; NF-κB: Nuclear factor kappa B; NGF: Nerve growth factor; NO: Nitric oxide; NOS: Nitric oxide synthase; Nrf2: Nuclear transcription factor E2-related factor 2; O2•–: Superoxide; PI3K/Akt: Phosphoinositol-3-kinase/Akt kinase; PYK2/CAK: Proline - rich tyrosine kinase 2/cell adhesion kinase; RAS: Renin-angiotensin system; RhoA: Small G-protein RhoA; RNS: Reactive nitrogen species; ROCK: Rho-associated protein kinase (serine/threonine kinase); ROS: Reactive oxygen species; RVLM: Rostral ventrolateral medulla; SHR: Spontaneously hypertensive rats.

## Competing interests

The authors declare that they have no competing interests.

## Authors´ contributions

MM, ID, MB, and JYHC collected information and prepared manuscript. MM and JYHC prepared the figures, ID, MB and JYHC finalized the manuscript. All authors read and approved the final manuscript.
